# Supplementary material for: Polypharmacology guided drug repositioning approach for SARS-CoV2
Source: PLoS One. 2023 Aug 9;18(8):e0289890. doi: 10.1371/journal.pone.0289890 (PMC10411734; doi:10.1371/journal.pone.0289890)
Supplement: S1 File — (DOCX) [file pone.0289890.s001.docx]

**Supporting Information**

**Polypharmacology Guided Drug Repositioning Approach for SARS-CoV2**

Esther Jamir*^1,2^, Himakshi Sarma^1^, Lipsa Priyadarsinee^1,2^, Kikrusenuo Kiewhuo^1,2^, Selvaraman Nagamani^1,2^ and G. Narahari Sastry*^1,2^

^1^Advanced Computation and Data Sciences Division, CSIR – North East Institute of Science and Technology, Jorhat – 785006, Assam, India

^2^Academy of Scientific and Innovative Research (AcSIR), Ghaziabad, India

*Corresponding Author: [gnsastry@gmail.com](mailto:gnsastry@gmail.com); [gnsastry@neist.res.in](mailto:gnsastry@neist.res.in); essjmr@gmail.com


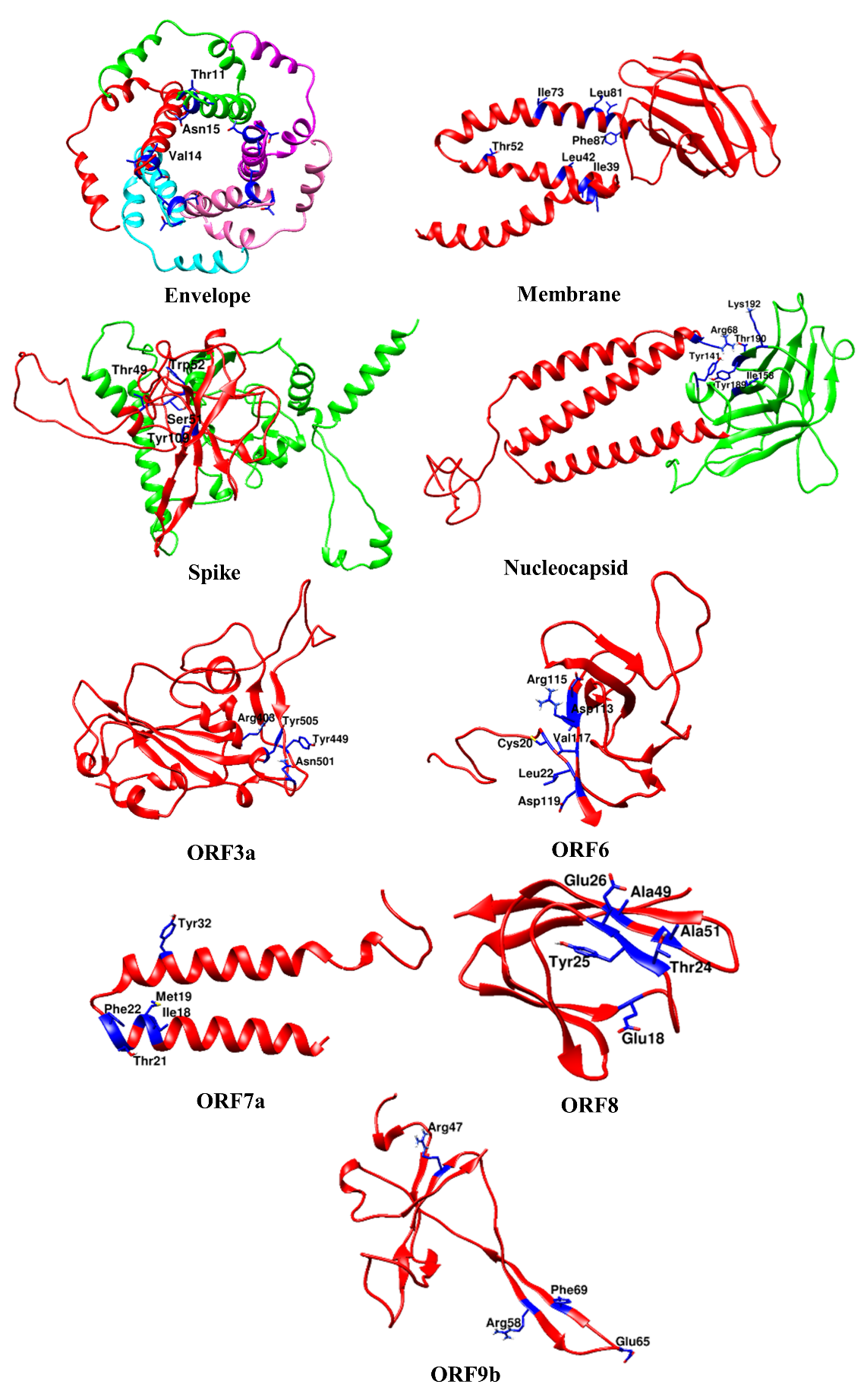


**Fig S1a.** 3D structure of structural (Envelope, Membrane, Spike, Nucleocapsid) and accessory (ORF3a, ORF6, ORF7a, ORF8, ORF9b) SARS CoV2 targets along with the active sites of the protein.


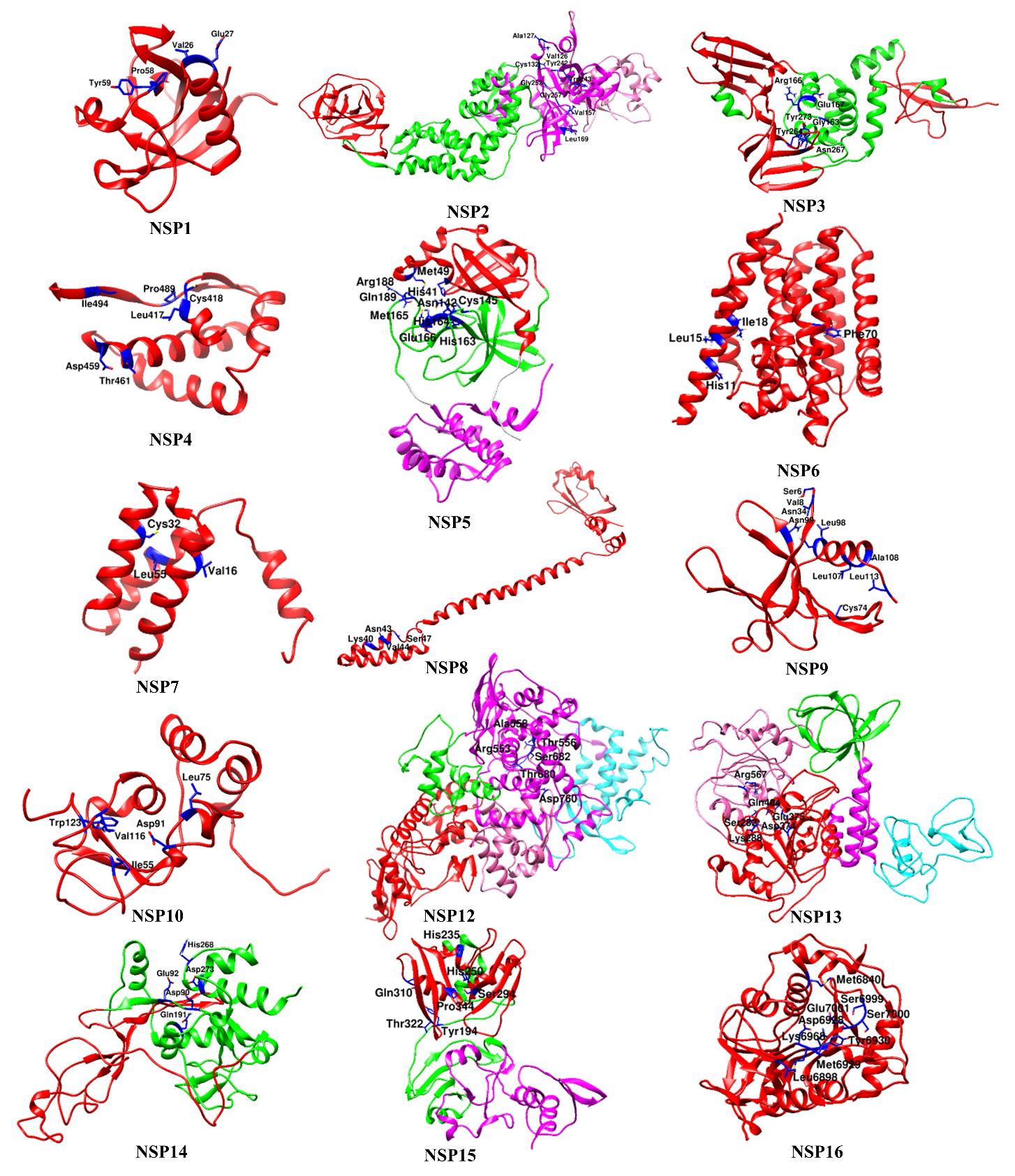


**Fig S1b.** 3D structure of non-structural (NSP1, NSP2, NSP3, NSP4, NSP5, NSP6, NSP7, NSP8, NSP9, NSP10, NSP12, NSP13, NSP14, NSP15, NSP16) SARS CoV2 targets along with the active sites of the protein.


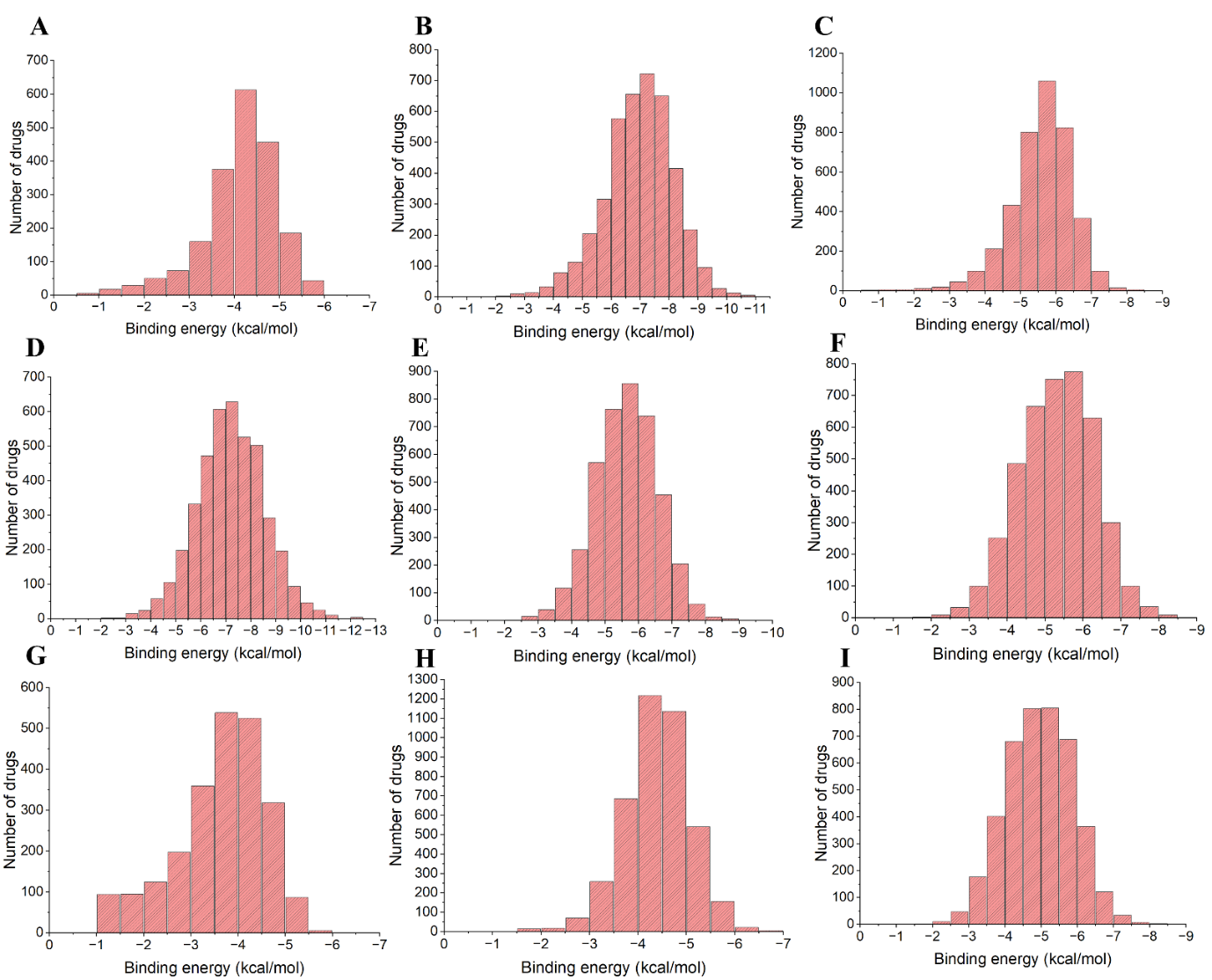


**Fig S2a.** Distribution of binding scores of 4193 compounds against 4 structural and 5 accessory SARS-CoV-2 targets namely A) Envelope, B) Membrane, C) Spike and D) Nucleoprotein, E) ORF3a, F) ORF6, G) ORF7a, H) ORF8 and I) ORF9b.


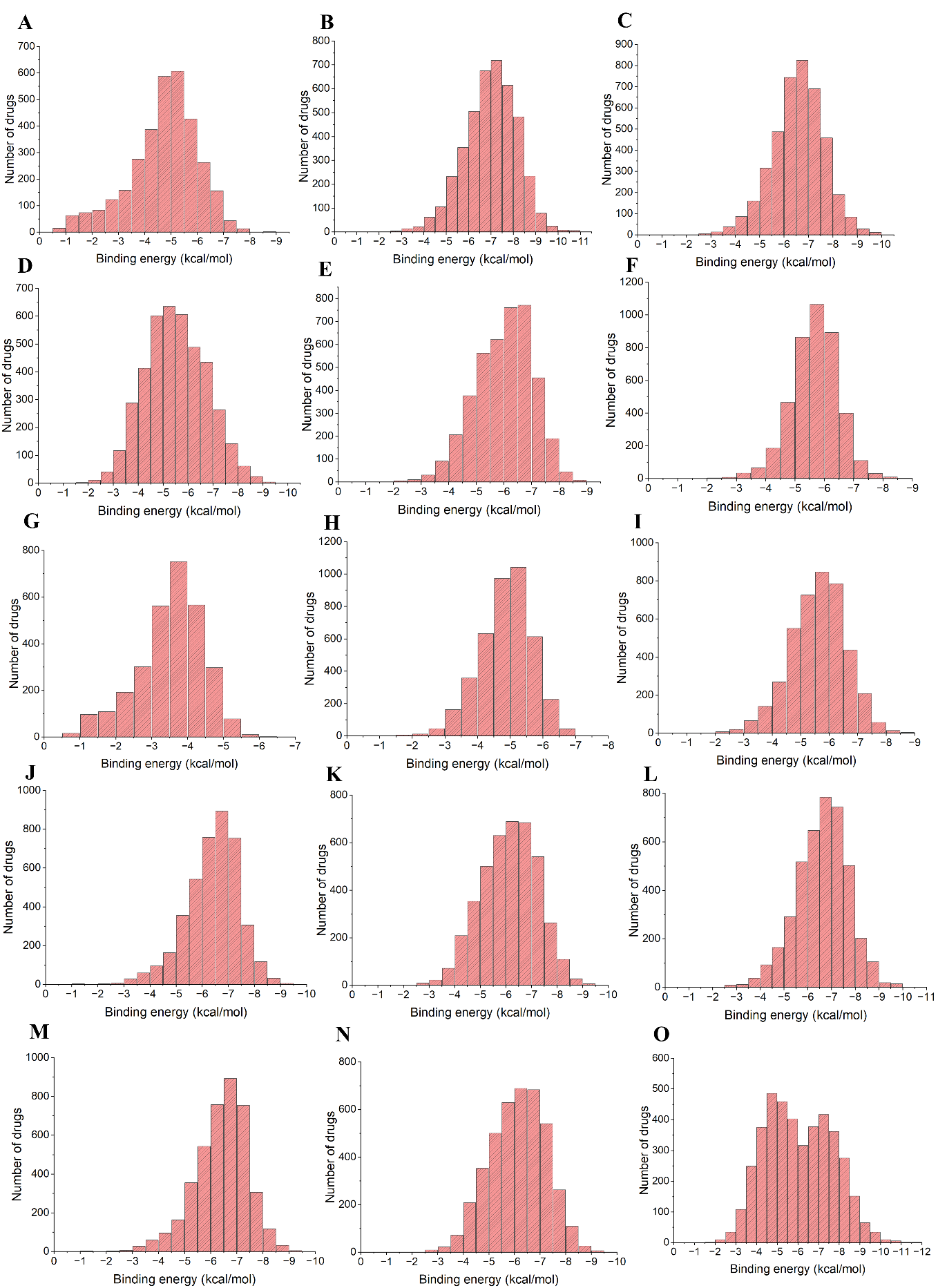


**Fig S2b.** Distribution of binding score of 4193 compounds against 15 non-structural SARS-CoV-2 targets namely A) NSP1 B) NSP2, C) NSP3 D) NSP4, E) NSP5, F) NSP6, G) NSP7, H) NSP8, I) NSP9, J) NSP10, K) NSP12, L) NSP13, M) NSP14, N) NSP15, O) NSP16.


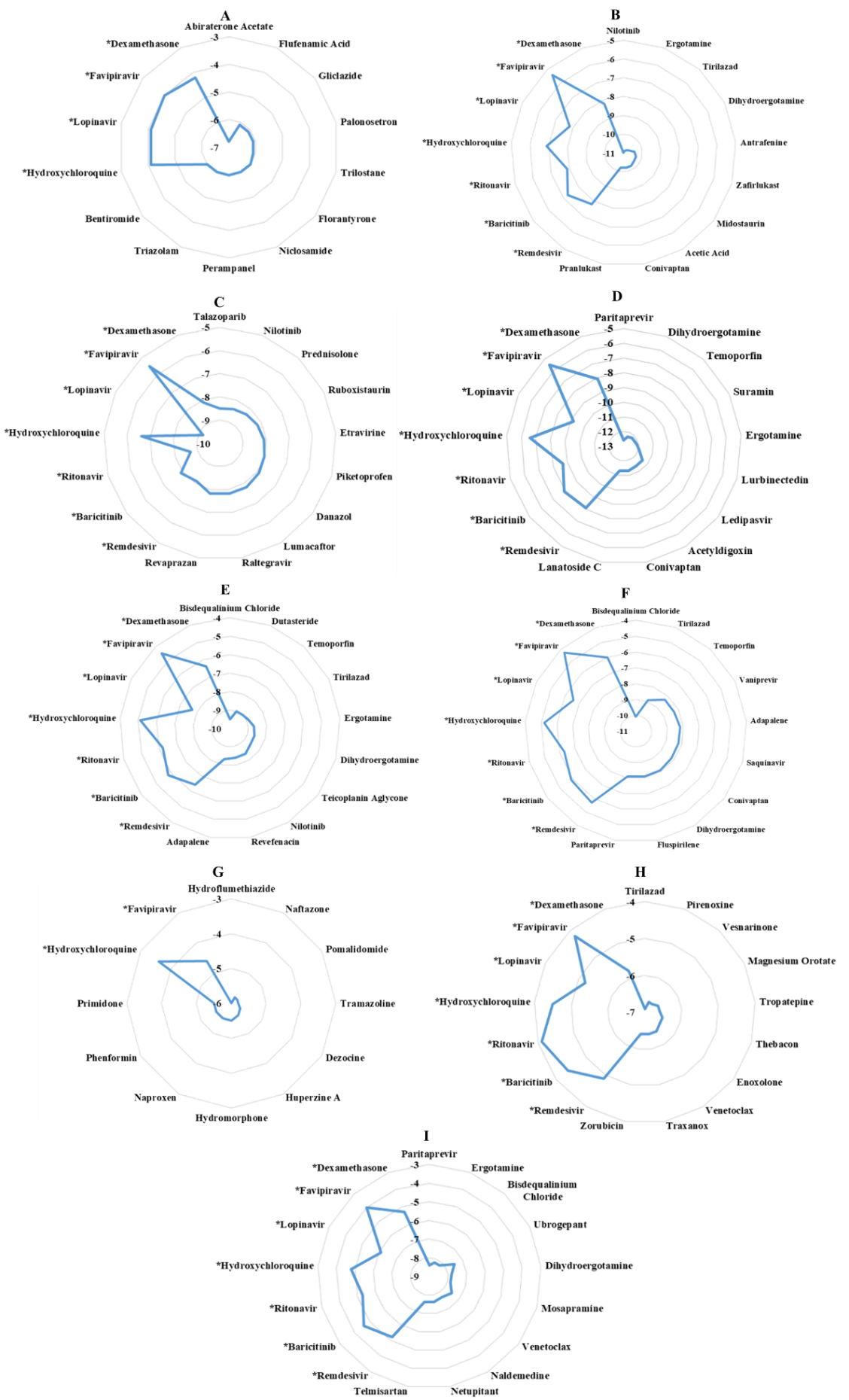


**Fig S3a.** The binding score distribution of top 10 FDA approved drugs against SARS-CoV2 targets that are structural proteins namely A) Envelope B) Membrane C) Spike D) Nucleoprotein and accessory proteins E) ORF3a F) ORF6 G) ORF7a H) ORF8 and I) ORF9. The drugs which are approved and under clinical trials for COVID-19 showing binding affinity against the selected targets have been highlighted (*) in the graph.

**
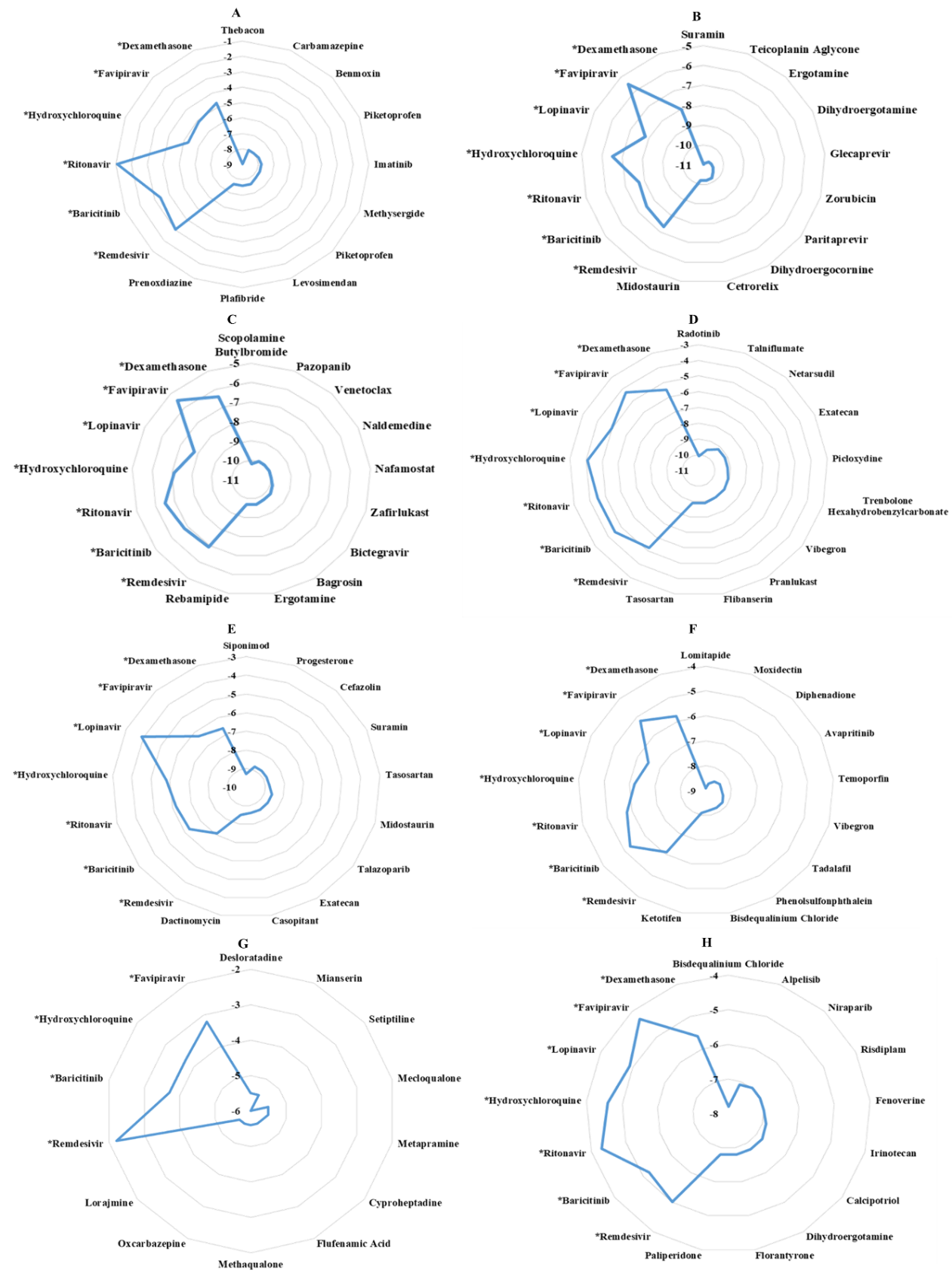
**

**Fig S3b.** The binding score distribution of top 10 FDA approved drugs against SARS-CoV2 targets that are non-structural proteins namely A) NSP1 B) NSP2 C) NSP3 D) NSP4 E) NSP5 F) NSP6 G) NSP7 and H) NSP8. The drugs which are approved and under clinical trials for COVID-19 showing binding affinity towards the selected targets have been highlighted (*) in the graph.


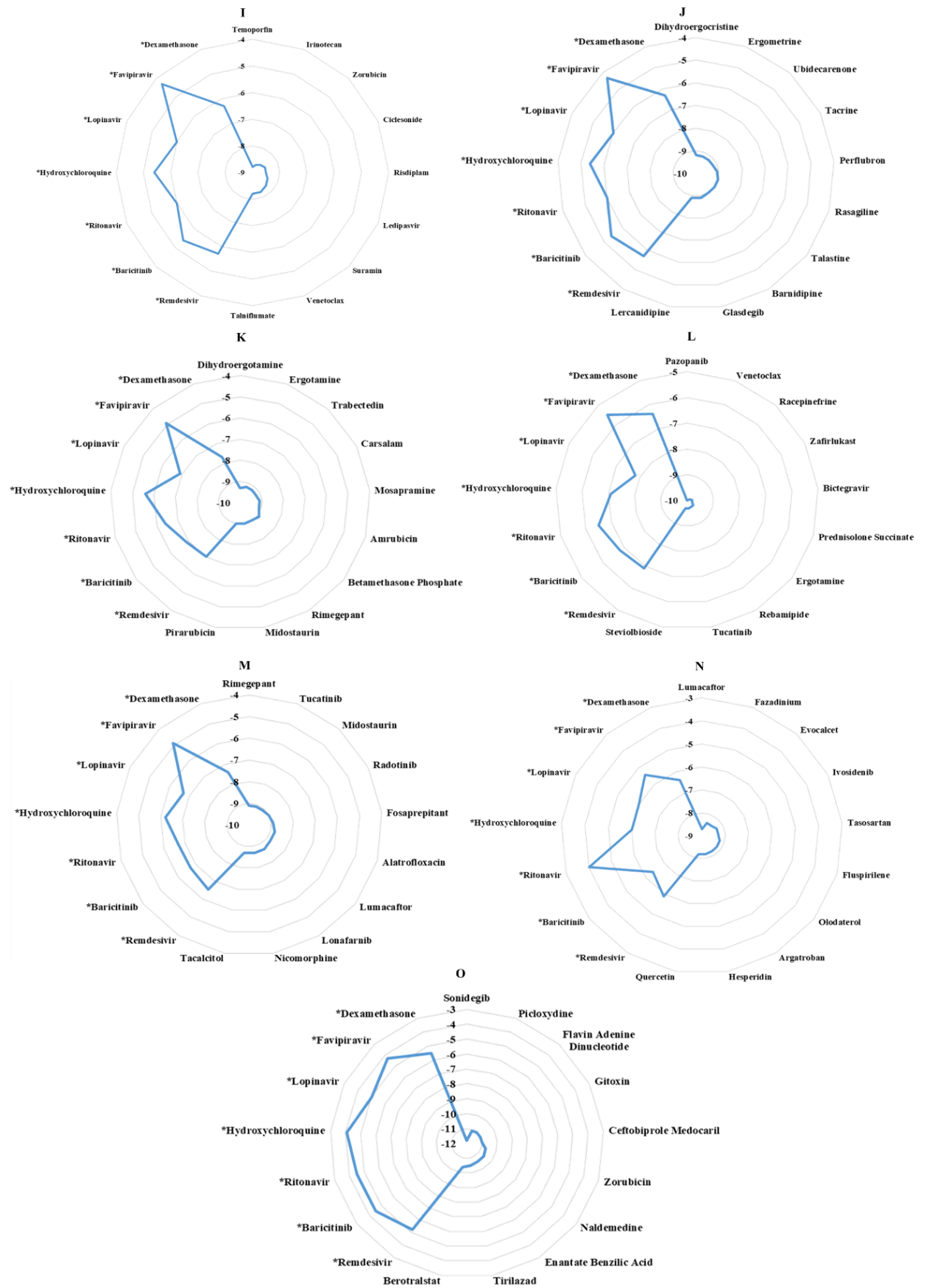


**Fig S3c.** The binding score distribution of top 10 FDA approved drug against the SARS-CoV2 targets that are non-structural namely I) NSP 9 J) NSP10 K) NSP12 L) NSP13 M) NSP14 N) NSP15 and O) NSP26. The drugs which are approved and under clinical trials for COVID-19 showing binding affinity towards the selected targets have been highlighted (*) in the graph.

**Fig S4a.** 2D interaction of drugs interaction with 8 SARS-CoV2 proteins. (a) Dihydroergotamine and (b) Ergotamine with its multiple SARS-CoV2 targets which are listed respectively. The 2D interactions shows the influence of various bond formations involved in the protein-ligand complex.

**Fig S4b.** 2D interaction of drugs interaction with 5 SARS-CoV2 proteins. (a) Bisdequalinium chloride, (b) Midostaurin, (c) Temoporfin, (d) Tirilazad and (e) Venetoclax with its multiple SARS-CoV2 targets. The 2D interactions shows the influence of various bond formations involved in the protein-ligand complex.

**Fig S4c.** 2D interaction of drugs interaction with 4 SARS-CoV2 proteins. (a) Paritaprevir, (b) Zorubicin, and (c) Suramin with its multiple SARS-CoV2 targets. The 2D interactions shows the influence of various bond formations involved in the protein-ligand complex.

**Fig S4d.** 2D interaction of drugs interaction with 3 SARS-CoV2 proteins. (a) Conivaptan, (b) Lumacaftor, (c) Naldemedine, (d) Nilotinib, (e) Tasosartan, (f) Zafirlukast The 2D interactions shows the influence of various bond formations involved in the protein-ligand complex.

**Table S1.** The grid box coordinates and size parameters used in AutoDock Vina for the 24 SARS-CoV2 proteins.

| **Coordinates** |  | **Proteins** | | | | | | | | | | | |
| --- | --- | --- | --- | --- | --- | --- | --- | --- | --- | --- | --- | --- | --- |
|  |  | **Envelope** | **Membrane** | **Spike** | **Nucleoprotein** | **ORF3a** | **ORF6** | **ORF7a** | **ORF8** | **ORF9b** | **NSP1** | **NSP2** | **NSP3** |
| Center (Å) | x | 9.8 | 5.278 | -37.399 | 78.331 | 79.192 | 55.813 | -25.319 | 43.543 | 9.372 | 5.508 | 111.543 | 1.569 |
|  | y | 2.974 | -6.826 | 20.878 | 85.622 | 82.982 | 57.921 | 18.671 | 34.034 | -6.727 | -12.859 | 117.089 | 7.464 |
|  | z | -6.155 | 2.919 | 7.558 | 79.184 | 54.222 | 60.902 | 17.793 | 118.323 | -10.841 | 20.384 | 106.244 | 34.271 |
|  |  |  |  |  |  |  |  |  |  |  |  |  |  |
| Box Size (Å) | x | 14 | 28 | 20 | 34 | 32 | 52 | 18 | 16 | 20 | 18 | 48 | 68 |
|  | y | 10 | 24 | 16 | 30 | 30 | 22 | 14 | 28 | 40 | 16 | 32 | 54 |
|  | z | 16 | 30 | 16 | 40 | 24 | 40 | 14 | 16 | 38 | 10 | 42 | 50 |
|  |  |  |  |  |  |  |  |  |  |  |  |  |  |
|  |  | **NSP4** | **NSP5** | **NSP6** | **NSP7** | **NSP8** | **NSP9** | **NSP10** | **NSP12** | **NSP13** | **NSP14** | **NSP15** | **NSP16** |
| Center (Å) | x | -2.583 | 15.001 | 57.093 | -20.321 | 174.111 | 44.798 | -40.598 | 89.486 | -57.825 | -27.091 | -51.175 | 86.941 |
|  | y | -2.998 | -16.402 | 62.528 | -27.202 | 125.217 | -10.821 | 5.367 | 92.499 | 2.01 | -1.648 | 23.763 | 24.217 |
|  | z | 15.26 | 22.172 | 62.16 | -5.998 | 171.567 | 19.821 | 25.819 | 96.486 | 26.436 | 1.615 | 30.092 | 30.756 |
|  |  |  |  |  |  |  |  |  |  |  |  |  |  |
| Box Size (Å) | x | 20 | 26 | 38 | 12 | 16 | 26 | 28 | 26 | 28 | 28 | 20 | 24 |
|  | y | 16 | 28 | 28 | 10 | 18 | 32 | 20 | 34 | 24 | 24 | 26 | 26 |
|  | z | 22 | 22 | 26 | 16 | 14 | 30 | 18 | 34 | 20 | 20 | 26 | 28 |

**Table S2:** List of docking scores of top 10 drugs against 24 SARS-CoV2 targets obtained using Autodock Vina, SwissDock, iGEMDOCKER, and MT-DTI.

| **Proteins** | **Drug Name** | **Autodock vina (kcal/mol)** | **SwissDock (kcal/mol)** | **iGemDock (kcal/mol)** | **MT-DTI** |
| --- | --- | --- | --- | --- | --- |
| **Envelope** | Abiraterone Acetate | -6.8 | -8.2 | 28.3 | 54.3 |
|  | Flufenamic Acid | -6.1 | -8.4 | -5.3 | 53.1 |
|  | Gliclazide | -6.1 | -8.2 | -5.7 | 48.3 |
|  | Palonosetron | -6.1 | -7.8 | -6 | 40.2 |
|  | Trilostane | -6.1 | -8 | -6 | 39.2 |
|  | Florantyrone | -6 | -7.9 | -6.2 | 42.9 |
|  | Niclosamide | -6 | -7.9 | 13.6 | 45.0 |
|  | Perampanel | -6 | -8 | -7.2 | 39.5 |
|  | Triazolam | -6 | -7.9 | -6.6 | 45.1 |
|  | Bentiromide | -6 | -7.7 | -6.5 | 48.1 |
|  |  |  |  |  |  |
| **Membrane** | Nilotinib | -11 | -7.9 | -8.7 | 75.2 |
|  | Ergotamine | -10.9 | -8.1 | -7.6 | 71.9 |
|  | Tirilazad | -10.8 | -7.5 | -9.1 | 70.1 |
|  | Dihydroergotamine | -10.7 | -8.8 | -6.7 | 68.3 |
|  | Antrafenine | -10.4 | -8.8 | -7.7 | 65.1 |
|  | Zafirlukast | -10.3 | -7.8 | -8.6 | 62.4 |
|  | Midostaurin | -10.3 | -7.8 | -7.3 | 63.2 |
|  | Acetic Acid | -10.2 | -8.5 | -8.4 | 62.1 |
|  | Conivaptan | -10.2 | -8.3 | -7.5 | 60.1 |
|  | Pranlukast | -10.2 | -7.6 | -7.6 | 61.9 |
|  |  |  |  |  |  |
| **Spike** | Talazoparib | -8.5 | -6.9 | -7.4 | 52.1 |
|  | Nilotinib | -8.4 | -7.1 | -7.7 | 43.6 |
|  | Prednisolone | -8.3 | -7.2 | -6.3 | 46.4 |
|  | Ruboxistaurin | -8.2 | -5.9 | -7.4 | 48.5 |
|  | Etravirine | -8.1 | -7.3 | -6.8 | 42.5 |
|  | Piketoprofen | -8 | -6.8 | -6.4 | 41.3 |
|  | Danazol | -7.9 | -5.8 | -6.5 | 39.4 |
|  | Lumacaftor | -7.8 | -7.4 | -6.5 | 32.4 |
|  | Raltegravir | -7.8 | -6.5 | -7.7 | 30.5 |
|  | Revaprazan | -7.8 | -6.8 | -6.2 | 30.5 |
|  |  |  |  |  |  |
| **Nuceloprotein** | Paritaprevir | -12.6 | -8.6 | -7.6 | 64.5 |
|  | Dihydroergotamine | -12.3 | -8.2 | -7.3 | 62.1 |
|  | Temoporfin | -12.2 | -8.2 | -9.6 | 60.5 |
|  | Suramin | -12.2 | -7.8 | -9.9 | 65.3 |
|  | Ergotamine | -12.1 | -8.2 | -7.4 | 59.5 |
|  | Lurbinectedin | -11.9 | -7.9 | -8.5 | 52.1 |
|  | Ledipasvir | -11.4 | -7.8 | -7.2 | 55.6 |
|  | Acetyldigoxin | -11.4 | -7.7 | -7.3 | 59.6 |
|  | Conivaptan | -11.3 | -8.2 | -7.7 | 58.6 |
|  | Lanatoside C | -11.3 | -8.1 | -8.3 | 52.1 |
|  |  |  |  |  |  |
| **NSP1** | Thebacon | -9 | -6.9 | -6.9 | 48.6 |
|  | Carbamazepine | -8 | -7.11 | -5.9 | 42.6 |
|  | Benmoxin | -8 | -6.8 | -6.1 | 47.9 |
|  | Piketoprofen | -7.9 | -6.8 | -6 | 48.6 |
|  | Imatinib | -7.8 | -6.8 | -7.3 | 41.6 |
|  | Methysergide | -7.8 | -6.7 | -5.7 | 40.8 |
|  | Scopolamine Butylbromide | -7.8 | -6.8 | -6.4 | 35.6 |
|  | Levosimendan | -7.6 | -6.8 | -6.4 | 32.4 |
|  | Plafibride | -7.6 | -6.7 | -6.3 | 38.6 |
|  | Prenoxdiazine | -7.6 | -6.6 | -6.1 | 39.4 |
|  |  |  |  |  |  |
| **NSP2** | Suramin | -11 | -9.9 | -6.5 | 60.5 |
|  | Teicoplanin Aglycone | -11 | -11.5 | -5.5 | 62.6 |
|  | Ergotamine | -10.9 | -11.2 | -7.4 | 59.6 |
|  | Dihydroergotamine | -10.7 | -10.1 | -8.1 | 58.66 |
|  | Glecaprevir | -10.6 | -12.5 | -6.5 | 59.62 |
|  | Zorubicin | -10.5 | -8.8 | -7.9 | 60.29 |
|  | Paritaprevir | -10.4 | -10.3 | -5.6 | 48.6 |
|  | Dihydroergocornine | -10.2 | -10.8 | -7.7 | 49.6 |
|  | Cetrorelix | -10.2 | -10.5 |  | 52.6 |
|  | Midostaurin | -10.2 | -10.8 | -5.7 | 59.6 |
|  |  |  |  |  |  |
| **NSP3** | Scopolamine Butylbromide | -10.2 | -7.2 | -6.7 | 47.6 |
|  | Pazopanib | -10 | -6.9 | -6 | 49.6 |
|  | Venetoclax | -10 | -8.2 | 1.4 | 42.9 |
|  | Naldemedine | -10 | -7.1 | -7 | 45.9 |
|  | Nafamostat | -10 | -7 | -6.7 | 47.8 |
|  | Zafirlukast | -9.9 | -7.1 | -8.2 | 48.9 |
|  | Bictegravir | -9.8 | -7.6 | -8.9 | 49.6 |
|  | Bagrosin | -9.8 | -8.1 | -7.6 | 52.9 |
|  | Ergotamine | -9.7 | -7.1 | -8 | 50.9 |
|  | Rebamipide | -9.7 | -8.3 | -6.9 | 48.6 |
|  |  |  |  |  |  |
| **NSP4** | Radotinib | -10.1 | -7.8 | -6.5 | 52.6 |
|  | Talniflumate | -9.6 | -7.9 | -8.9 | 50.3 |
|  | Netarsudil | -9.2 | -8.4 | -8.7 | 49.6 |
|  | Exatecan | -9.2 | -8 | -7.6 | 48.1 |
|  | Picloxydine | -9.2 | -8.1 | -9.2 | 47.6 |
|  | Trenbolone Hexahydrobenzylcarbonate | -9.1 | -8 | -9.6 | 48.6 |
|  | Vibegron | -9 | -8.1 | -7.6 | 42.6 |
|  | Pranlukast | -9 | -8.3 | -9.2 | 40.6 |
|  | Flibanserin | -8.9 | -8.1 | -7 | 39.6 |
|  | Tasosartan | -8.9 | -8.1 | -7.3 | 39.6 |
|  |  |  |  |  |  |
| **NSP5** | Siponimod | -9.3 | -10 | -6.8 | 68.6 |
|  | Progesterone | -8.8 | -8.3 | -5.3 | 52.6 |
|  | Cefazolin | -8.8 | -9.7 | -5.3 | 51.6 |
|  | Suramin | -8.8 | -8.9 | -8.6 | 49.6 |
|  | Tasosartan | -8.8 | -9.6 | -6.3 | 48.6 |
|  | Midostaurin | -8.6 | -8.2 | -7.5 | 42.6 |
|  | Talazoparib | -8.6 | -9.1 | -7.8 | 40.6 |
|  | Exatecan | -8.6 | -9.7 | -7.4 | 41.6 |
|  | Casopitant | -8.6 | -8.4 | -6.2 | 42.6 |
|  | Dactinomycin | -8.5 | -9.1 | -6.2 | 49.4 |
|  |  |  |  |  |  |
| **NSP6** | Lomitapide | -8.9 | -7.3 | -5.5 | 45.6 |
|  | Moxidectin | -8.7 | -7.3 | -6.5 | 42.6 |
|  | Diphenadione | -8.5 | -7.5 | -5.7 | 41.6 |
|  | Avapritinib | -8.4 | -7.3 | -7.8 | 46.2 |
|  | Temoporfin | -8.4 | -7.5 | -7.3 | 47.2 |
|  | Vibegron | -8.3 | -7.3 | -6.4 | 40.6 |
|  | Tadalafil | -8.2 | -7.5 | -7.2 | 39.8 |
|  | Phenolsulfonphthalein | -8.2 | -7.2 | -6 | 38.6 |
|  | Bisdequalinium Chloride | -8.2 | -7.3 | -6.8 | 37.2 |
|  | Ketotifen | -8.1 | -8.2 | -6.3 | 36.2 |
|  |  |  |  |  |  |
| **NSP7** | Setiptiline | -6 | -6.2 | -4.3 | 28.6 |
|  | Cyproheptadine | -5.6 | -6.7 | -5.7 | 35.2 |
|  | Flufenamic Acid | -5.6 | -6.4 | -5 | 29.6 |
|  | Methaqualone | -5.6 | -6.7 | -4.7 | 30.3 |
|  | Oxcarbazepine | -5.6 | -6.3 | -4 | 32.6 |
|  | Lorajmine | -5.6 | -6.4 | -5 | 35.3 |
|  | Mecloqualone | -5.5 | -6.2 | -4.6 | 36.7 |
|  | Metapramine | -5.5 | -6.3 | -4.5 | 38.6 |
|  | Desloratadine | -5.5 | -6.5 | -5.8 | 29.3 |
|  | Mianserin | -5.5 | -6.2 | -5.3 | 28.6 |
|  |  |  |  |  |  |
| **NSP8** | Bisdequalinium Chloride | -7.8 | -6.8 | -4.1 | 62.6 |
|  | Alpelisib | -7.1 | -7.6 | -5.5 | 58.6 |
|  | Niraparib | -7 | -7.1 | -5 | 59.3 |
|  | Risdiplam | -7 | -6.1 | -5.9 | 62.5 |
|  | Fenoverine | -7 | -6.9 | -6 | 61.3 |
|  | Irinotecan | -6.9 | -6.4 | -5.8 | 60.8 |
|  | Calcipotriol | -6.8 | -6.8 | -4.9 | 51.6 |
|  | Dihydroergotamine | -6.8 | -6.2 | -4.6 | 50.8 |
|  | Florantyrone | -6.8 | -6.4 | -6 | 55.6 |
|  | Paliperidone | -6.8 | -6.6 | -6.4 | 59.2 |
|  |  |  |  |  |  |
| **NSP9** | Temoporfin | -8.8 | -7.9 | -8.4 | 47.6 |
|  | Irinotecan | -8.7 | -7.3 | -7.2 | 42.6 |
|  | Zorubicin | -8.6 | -7.4 | -8.4 | 45.9 |
|  | Ciclesonide | -8.5 | -7.4 | -6.9 | 48.3 |
|  | Risdiplam | -8.5 | -7.5 | -7.4 | 49.3 |
|  | Ledipasvir | -8.4 | -8.2 | -8 | 50.6 |
|  | Suramin | -8.3 | -7.3 | -9.9 | 47.2 |
|  | Venetoclax | -8.2 | -8.41 | -7.5 | 40.2 |
|  | Talniflumate | -8.2 | -7.2 | -6.8 | 39.6 |
|  | Quinbolone | -8.2 | -7.8 | -6.2 | 38.6 |
|  |  |  |  |  |  |
| **NSP10** | Dihydroergocristine | -9.2 | -9 | -9.3 | 52.6 |
|  | Ergometrine | -9.2 | -8.3 | -6.3 | 51.3 |
|  | Ubidecarenone | -9.2 | -7.7 | -5.1 | 58.3 |
|  | Tacrine | -9.2 | -7.9 | -6.2 | 49.3 |
|  | Perflubron | -9.1 | -7.5 | -5.7 | 48.1 |
|  | Rasagiline | -9 | -8 | -5.4 | 47.2 |
|  | Talastine | -9 | -8.3 | -6.9 | 45.3 |
|  | Barnidipine | -9 | -8.2 | -7.1 | 44.3 |
|  | Glasdegib | -8.9 | -8.2 | -7.3 | 43.6 |
|  | Lercanidipine | -8.9 | -8.3 | -6.8 | 39.2 |
|  |  |  |  |  |  |
| **NSP12** | Dihydroergotamine | -9.3 | -7.8 | -7.4 | 55.6 |
|  | Ergotamine | -9.2 | -7.5 | -7.4 | 54.6 |
|  | Trabectedin | -9.2 | -6.8 | -5.8 | 53.1 |
|  | Carsalam | -9.2 | -8 | -6.3 | 52.6 |
|  | Mosapramine | -9.1 | -6.9 | -5.9 | 49.2 |
|  | Amrubicin | -9.1 | -7 | -7.2 | 58.6 |
|  | Rimegepant | -9 | -7.8 | -6.3 | 42.6 |
|  | Midostaurin | -9 | -7.6 | -8 | 40.1 |
|  | Pirarubicin | -9 | -6.8 | -5.8 | 45.6 |
|  | Betamethasone Phosphate | -8.9 | -8.6 | -6.4 | 48.1 |
|  |  |  |  |  |  |
| **NSP13** | Pazopanib | -10 | -8 | -7.8 | 61.2 |
|  | Venetoclax | -10 | -7.1 | -8 | 60.2 |
|  | Racepinefrine | -10 | -7.7 | -6.3 | 58.1 |
|  | Zafirlukast | -9.9 | -7.7 | -7.2 | 56.6 |
|  | Bictegravir | -9.8 | -8.8 | -6.4 | 59.6 |
|  | Prednisolone Succinate | -9.8 | -7.6 | -6.4 | 61.2 |
|  | Ergotamine | -9.7 | -7.9 | -8.7 | 54.6 |
|  | Rebamipide | -9.7 | -7.3 | -7.3 | 53.2 |
|  | Tucatinib | -9.7 | -7.2 | -7.8 | 52.6 |
|  | Steviolbioside | -9.7 | -7.7 | -9.7 | 58.1 |
|  |  |  |  |  |  |
| **NSP14** | Rimegepant | -9.1 | -7.7 | -5.8 | 48.1 |
|  | Tucatinib | -9.1 | -8.7 | -7.8 | 49.1 |
|  | Midostaurin | -9.1 | -8.3 | -8.4 | 51.1 |
|  | Radotinib | -9 | -7.4 | -9 | 50.1 |
|  | Fosaprepitant | -8.9 | -8.5 | -7 | 53.1 |
|  | Alatrofloxacin | -8.8 | -7.8 | -9 | 54.1 |
|  | Lumacaftor | -8.8 | -7.8 | -8.3 | 53.1 |
|  | Lonafarnib | -8.7 | -7.9 | -7 | 52.1 |
|  | Nicomorphine | -8.7 | -7.5 | -8.6 | 51.6 |
|  | Tacalcitol | -8.7 | -7.7 | -6.8 | 47.1 |
|  |  |  |  |  |  |
| **NSP15** | Lumacaftor | -8.7 | -7.5 | -6.6 | 56.1 |
|  | Fazadinium | -8.4 | -7.5 | -7.5 | 54.1 |
|  | Evocalcet | -8.4 | -8.3 | -6.7 | 53.1 |
|  | Ivosidenib | -8.3 | -8.2 | -7.6 | 56.1 |
|  | Tasosartan | -8.3 | -7.9 | -7.4 | 53.2 |
|  | Fluspirilene | -8.2 | -7.6 | -8.2 | 54.2 |
|  | Olodaterol | -8.2 | -7.4 | -7.4 | 48.5 |
|  | Argatroban | -8.2 | -7.5 | -6.5 | 49.6 |
|  | Hesperidin | -8.2 | -7.5 | -7.6 | 50.1 |
|  | Quercetin | -8.2 | -6.2 | -7.5 | 52.0 |
|  |  |  |  |  |  |
| **NSP16** | Sonidegib | -11.8 | -7.5 | -6 | 61.6 |
|  | Picloxydine | -11.1 | -7.4 | -10.6 | 63.1 |
|  | Flavin Adenine Dinucleotide | -11 | -7.4 | -6.1 | 59.1 |
|  | Gitoxin | -11 | -8.2 | -7.8 | 52.1 |
|  | Ceftobiprole Medocaril | -11 | -7.8 | -8.3 | 55.1 |
|  | Zorubicin | -10.7 | -8 | -8.9 | 54.6 |
|  | Naldemedine | -10.6 | -7.3 | -7.5 | 52.6 |
|  | Enantate Benzilic Acid | -10.6 | -8.9 | -7.1 | 53.6 |
|  | Tirilazad | -10.5 | -7.1 | -7 | 54.6 |
|  | Berotralstat | -10.4 | -8 | -7.8 | 55.5 |
|  |  |  |  |  |  |
| **ORF3a** | Bisdequalinium Chloride | -9.5 | -7.7 | -7.2 | 48.2 |
|  | Dutasteride | -9 | -7.1 | -7.9 | 47.6 |
|  | Temoporfin | -9 | -7.4 | -10.4 | 48.6 |
|  | Tirilazad | -8.9 | -7.6 | -5.7 | 49.6 |
|  | Ergotamine | -8.7 | -6.8 | -7 | 49.2 |
|  | Dihydroergotamine | -8.6 | -7.2 | -7.6 | 48.6 |
|  | Teicoplanin Aglycone | -8.6 | -6.6 | -5.3 | 51.6 |
|  | Nilotinib | -8.4 | -6.8 | -7.1 | 48.6 |
|  | Revefenacin | -8.4 | -6.6 | -8 | 49.6 |
|  | Adapalene | -8.3 | -6.8 | -6.3 | 41.6 |
|  |  |  |  |  |  |
| **ORF6** | Bisdequalinium Chloride | -10.1 | -6.7 | -6.5 | 52.6 |
|  | Tirilazad | -8.9 | -6.1 | -6.5 | 51.6 |
|  | Temoporfin | -8.3 | -7 | -5.6 | 53.5 |
|  | Vaniprevir | -8.3 | -6.1 | -3.1 | 52.3 |
|  | Adapalene | -8.2 | -8.1 | -6.3 | 54.6 |
|  | Saquinavir | -8.2 | -6.8 | -8.4 | 52.3 |
|  | Conivaptan | -8.2 | -6.6 | -6.1 | 50.6 |
|  | Dihydroergotamine | -8.1 | -7.7 | -8.2 | 48.6 |
|  | Fluspirilene | -8.1 | -7 | -6.9 | 49.1 |
|  | Paritaprevir | -8.1 | -6.2 | -4.5 | 47.5 |
|  |  |  |  |  |  |
| **ORF7a** | Hydroflumethiazide | -6 | -6 | -4.9 | 39.1 |
|  | Naftazone | -5.8 | -6 | -6 | 34.1 |
|  | Pomalidomide | -5.8 | -5.7 | -6.3 | 32.6 |
|  | Tramazoline | -5.8 | -5.7 | -6.2 | 32.1 |
|  | Dezocine | -5.7 | -6.3 | -4.7 | 31.6 |
|  | Huperzine A | -5.6 | -5.5 | -5.4 | 31 |
|  | Hydromorphone | -5.5 | -6 | -6.1 | 29.6 |
|  | Naproxen | -5.5 | -5.6 | -5.2 | 28.1 |
|  | Phenformin | -5.5 | -5.8 | -6.8 | 28.0 |
|  | Primidone | -5.5 | -5.9 | -4.9 | 27.0 |
|  |  |  |  |  |  |
| **ORF 8** | Tirilazad | -6.9 | -10.6 | -5.3 | 42.6 |
|  | Pirenoxine | -6.7 | -10.7 | -7.7 | 41.6 |
|  | Vesnarinone | -6.7 | -9.9 | -6.9 | 49.6 |
|  | Magnesium Orotate | -6.6 | -11.1 | -6.4 | 48.1 |
|  | Tropatepine | -6.6 | -10.6 | -7 | 41.2 |
|  | Thebacon | -6.5 | -10.1 | 2.6 | 46.6 |
|  | Enoxolone | -6.5 | -9.5 | -7 | 48.2 |
|  | Venetoclax | -6.4 | -10.3 | -7.4 | 49.4 |
|  | Traxanox | -6.4 | -10.7 | -7.8 | 42.6 |
|  | Zorubicin | -6.4 | -9.2 | -9.6 | 42.6 |
|  |  |  |  |  |  |
| **ORF 9b** | Paritaprevir | -8.4 | -7.1 | -3.9 | 59.4 |
|  | Ergotamine | -8.2 | -7.6 | -5.2 | 58.6 |
|  | Bisdequalinium Chloride | -8.2 | -6.9 | -6.2 | 58.1 |
|  | Dihydroergotamine | -7.8 | -7.8 | -7 | 59.2 |
|  | Mosapramine | -7.8 | -7.1 | -6.5 | 59.1 |
|  | Naldemedine | -7.7 | -6.9 | -5.7 | 57.1 |
|  | Netupitant | -7.6 | -7.31 | -7.2 | 54.2 |
|  | Telmisartan | -7.6 | -6.9 | -6.4 | 52.6 |
|  | Ubrogepant | -7.5 | -7.3 | -5 | 52.1 |
|  | Venetoclax | -7.5 | -7.2 | -7.3 | 50.1 |

**Table S3**: List of docking scores identified top multi targeting drugs against SARS-CoV2 targets obtained using Autodock Vina, SwissDock, iGEMDOCKER, and MT-DTI and free energy calculation scores obtained using FastDRH^1^.

| **Sl.no** | **Drugs** | **Targets** | **Autodock vina (kcal/mol)** | **SwissDock (kcal/mol)** | **iGemDock (kcal/mol)** | **MT-DTI** | **MM/PBSA (kcal/mol)** | **MM/GBSA (kcal/mol)** |
| --- | --- | --- | --- | --- | --- | --- | --- | --- |
| 1 | Bisdequalinium Chloride | NSP6 | -8.2 | -7.3 | -6.8 | 37.2 | -29.24 | -34.49 |
|  |  | NSP8 | -7.8 | -6.8 | -4.1 | 62.6 | -21.16 | -33.1 |
|  |  | ORF3a | -9.5 | -7.7 | -7.2 | 48.2 | -40.5 | -42.91 |
|  |  | ORF6 | -10.1 | -6.7 | -6.5 | 52.6 | -40.05 | -41.01 |
|  |  | ORF9b | -8.2 | -6.9 | -6.2 | 58.1 | -29.68 | -31.16 |
| 2 | Dihydroergotamine | Membrane | -10.7 | -8.8 | -6.7 | 68.3 | -46.89 | -54.68 |
|  |  | Nucleoprotein | -12.3 | -8.2 | -7.3 | 62.1 | -39.81 | -54.59 |
|  |  | NSP2 | -10.7 | -10.1 | -8.1 | 58.66 | -41.32 | -52.67 |
|  |  | NSP8 | -6.8 | -6.2 | -4.6 | 50.8 | -21.82 | -35.9 |
|  |  | NSP12 | -9.3 | -7.8 | -7.4 | 55.6 | -21.46 | -45.45 |
|  |  | ORF3a | -8.6 | -7.2 | -7.6 | 48.6 | -34.78 | -39.55 |
|  |  | ORF6 | -8.1 | -7.7 | -8.2 | 48.6 | -42.72 | -47.24 |
|  |  | ORF9b | -7.8 | -7.8 | -7 | 59.2 | -40.4 | -41.64 |
| 3 | Ergotamine | Membrane | -10.9 | -8.1 | -7.6 | 71.9 | -46.39 | -54.96 |
|  |  | Nucleoprotein | -12.1 | -8.2 | -7.4 | 59.5 | -41.57 | -55.85 |
|  |  | NSP2 | -10.9 | -11.2 | -7.4 | 59.6 | -41.89 | -52.41 |
|  |  | NSP3 | -9.7 | -7.1 | -8 | 50.9 | -36.7 | -47.8 |
|  |  | NSP12 | -9.2 | -7.5 | -7.4 | 54.6 | -19.5 | -43.36 |
|  |  | NSP13 | -9.7 | -7.9 | -8.7 | 54.6 | -15.75 | -51.43 |
|  |  | ORF3a | -8.7 | -6.8 | -7 | 49.2 | -38.94 | -43.71 |
|  |  | ORF9b | -8.2 | -7.6 | -5.2 | 58.6 | -40.3 | -43.2 |
| 4 | Midostaurin | Membrane | -10.3 | -7.8 | -7.3 | 63.2 | -42.65 | -48.35 |
|  |  | NSP2 | -10.2 | -10.8 | -5.7 | 59.6 | -33.02 | -50.1 |
|  |  | NSP5 | -8.6 | -8.2 | -7.5 | 42.6 | -17.00 | -39.00 |
|  |  | NSP12 | -9 | -7.6 | -8 | 40.1 | -12.77 | -43.6 |
|  |  | NSP14 | -9.1 | -8.3 | -8.4 | 51.1 | -2.21 | -40.76 |
| 5 | Temoporfin | Nucleoprotein | -12.2 | -8.2 | -9.6 | 60.5 | -46.71 | -59.9 |
|  |  | NSP6 | -8.4 | -7.5 | -7.3 | 47.2 | -44.45 | -48.36 |
|  |  | NSP9 | -8.8 | -7.9 | -8.4 | 47.6 | -34.25 | -42.49 |
|  |  | ORF3a | -9 | -7.4 | -10.4 | 48.6 | -45.91 | -49.69 |
|  |  | ORF6 | -8.3 | -7 | -5.6 | 53.5 | -30.02 | -42.49 |
| 6 | Tirilazad | Membrane | -10.8 | -7.5 | -9.1 | 70.1 | -26.28 | -30.38 |
|  |  | NSP16 | -10.5 | -7.1 | -7 | 54.6 | -19.26 | -32.76 |
|  |  | ORF3a | -8.9 | -7.6 | -5.7 | 49.6 | -18.75 | -40.75 |
|  |  | ORF6 | -8.9 | -6.1 | -6.5 | 51.6 | -38.45 | -43.75 |
|  |  | ORF8 | -6.9 | -10.6 | -5.3 | 42.6 | -15.01 | -34.88 |
| 7 | Venetoclax | NSP3 | -10 | -8.2 | 1.4 | 42.9 | -45.1 | -74.53 |
|  |  | NSP9 | -8.2 | -8.41 | -7.5 | 40.2 | -40.52 | -50.5 |
|  |  | NSP13 | -10 | -7.1 | -8 | 60.2 | -32.9 | -67.11 |
|  |  | ORF8 | -6.4 | -10.3 | -7.4 | 49.4 | -38.8 | -49.64 |
|  |  | ORF9b | -7.5 | -7.2 | -7.3 | 50.1 | -37.52 | -26.18 |

**Table S4.** The scaffold interaction with SARS-CoV2 proteins along with types of bonds and residues involved in the interaction.

| **Scaffold** | **Protein** | **Type of bond** | **Distance (Å)** | **Residue** |
| --- | --- | --- | --- | --- |
| 1-Benzazepine | Envelope | Pi-Alkyl | 5.28 | LEU18 |
|  |  | Carbon-Hydrogen Bond | 3.79 | ASN15 |
|  | Membrane | Pi-Pi Stacked | 5.86 | TRP22 |
|  |  | Unfavourable Bump | 2.36  2.12 | LEU48  PHE44 |
|  | NSP4 | Pi-Alkyl | 4.91 | CYS418 |
|  | NSP14 | Pi-Alkyl | 4.85  5.15 | ARG213  ARG205 |
|  |  | Conventional H-Bond | 2.02 | ARG213 |
|  |  | Unfavourable Bump | 2.35  1.74 | GLU204  ARG205 |
|  | ORF9b | Pi-Alkyl | 4.53 | PRO43 |
|  |  | Pi-Lone Pair | 2.69 | TYR42 |
|  |  | Pi-Sigma | 3.10 | LEU21 |
|  |  | Unfavourable Bump | 2.18  1.55  1.99  2.32  2.08 | VAL92  PRO43  TYR42  VAL23  LEU21 |
| 4,5,6,7,8,8a,9,10-Octahydro-2(3H)-phenanthrenone | Envelope | Alkyl | 5.05 | LEU18 |
|  |  | Unfavourable Bump | 1.13  2.36 | LEU18  ASN15 |
|  | Membrane | Pi-Alkyl | 3.59  3.87 | PHE19  TRP22 |
|  |  | Unfavourable Bump | 2.36  1.46 | ASN15  LEU18 |
|  | NSP4 | Alkyl | 5.14 | CYS418 |
|  | ORF9b | Unfavourable Bump | 2.13  2.27  2.22 | LYS40  VAL41  TYR42 |
|  |  | Pi-Alkyl | 4.99 | TYR42 |
|  |  | Alkyl | 3.67  3.62 | LYS40  VAL41 |
| 6,7,8,9-Tetrahydro-5H-cyclohepta[c]pyridine | Envelope | Unfavourable Bump | 2.21 | LEU18 |
|  | Membrane | Unfavourable Bump | 2.24  2.17 | TRP22  LEU48 |
|  | NSP4 | Alkyl | 5.33 | CYS418 |
|  | NSP5 | Carbon-Hydrogen Bond | 2.44 | GLU288 |
|  | NSP14 | Unfavourable Bump | 2.40  2.43 | GLU204  ARG205 |
|  | ORF9b | Unfavourable Bump | 2.32  2.34  2.41 | TYR42  PRO43  VAL92 |
|  |  | Carbon-Hydrogen Bond | 2.83 | TYR42 |
|  |  | Alkyl | 5.39 | LYS40 |
| Decalin | Envelope | Alkyl | 5.41 | LEU18 |
|  |  | Unfavourable Bump | 2.29 | LEU18 |
|  | Membrane | Alkyl | 5.49 | LEU45 |
|  |  | Unfavourable Bump | 2.32  1.96 | TRP22  LEU48 |
|  | NSP4 | Alkyl | 4.99 | CYS418 |
|  | NSP14 | Alkyl | 5.04 | ARG213 |
|  |  | Unfavourable Bump | 2.24 | GLU204 |
|  | ORF9b | Unfavourable Bump | 2.44  2.36  2.30 | VAL41  TYR42  VAL92 |
|  |  | Alkyl | 4.65  4.01 | LEU21  LYS40 |
| Gona-1,3,5(10)-trien-3-ol | Envelope | Alkyl | 5.07 | LEU18 |
|  |  | Unfavourable Bump | 2.44  2.11  2.42 | LEU18  ASN15  VAL14 |
|  | Membrane | Pi-Alkyl | 3.90  4.33 | PHE19  TRP22 |
|  |  | Unfavourable Bump | 2.24  2.42 | PHE19  TRP22 |
|  | NSP4 | Alkyl | 5.02 | CYS418 |
|  | NSP14 | Unfavourable Bump | 2.37 | GLU204 |
|  | ORF9b | Unfavourable Bump | 2.33  2.40  2.44 | LYS40  VAL41  TYR42 |
|  |  | Pi-alkyl | 4.55  4.33 | LYS40  VAL41 |
|  |  | Alkyl | 4.91 | TYR42 |
| leucoline | Envelope | Pi-Alkyl | 5.08 | LEU18 |
|  | Membrane | Pi-Sigma | 3.95 | LEU48 |
|  |  | Unfavourable Bump | 2.31 | LEU48 |
|  | NSP4 | Alkyl | 5.00 | CYS418 |
|  | NSP5 | Unfavourable Bump | 2.03 | GLU288 |
|  | NSP14 | Unfavourable Bump | 2.36  2.26 | GLU204  ARG205 |
|  |  | Pi-Alkyl | 5.26 | ARG213 |
|  | ORF9b | Unfavourable Bump | 2.46  2.27  2.14  1.97 | LEU21  TYR42  PRO43  VAL92 |
|  |  | Pi-Sigma | 3.19 | LEU21 |
|  |  | Pi-Lone Pair | 2.49 | TYR42 |
|  |  | Pi-alkyl | 5.38  4.50  5.08  3.67 | LEU21  VAL23  PRO43  VAL92 |

**Table S5:** List of top drugs interacting with multiple SARS-CoV2 targets with the interacting residues and amino acid residues involved in Hydrogen bon formation and Van der Waals bond.

| **Drug** | **Protein** | **Interacting residues** | **Hydrogen bond** | **Van der Waals bond** |
| --- | --- | --- | --- | --- |
| Dihydroergotamine | Membrane | ILE64, ILE67, THR52, LEU48, ALA72,TRP49 | ILE64, THR52, TRP49, LEU48 | - |
|  | NSP2 | LEU180, ALA184, TYR124, ASN183, GLN182, ILE104, PHE300, CYS326 | ASN183, GLN182 | - |
|  | NSP8 | - | - | - |
|  | NSP12 | ARG555, LYS551, ASP623, ARG624, LYS621 | ARG553, ARG624, ASP623 | - |
|  | Nulceoprotien | LEU121,GLY120,ALA119,GLU118,GLY116,PRO117,THR115,THR148,TYR86,ARG88,ARG89,ALA90 | THR115, ALA119 | - |
|  | ORF3a | PHE87,TRP45,ILE37 | - | - |
|  | ORF6 | PHE7, ILE11, GLU46, LYS48 | - | - |
|  | ORF9b | MET56, ILE74, ILE19,LEU54, VAL76, LEU52, VAL15, ILE44,VAL94,LEU21 | - | - |
| Ergotamine | Membrane | LEU45, THR52, TRP49, THR68, ALA72, VAL51, ILE67 | THR68 | - |
|  | NSP2 | CYS326, PHE300, SER299, ASN254, GLN182, ASN183, LEU180, ALA184, TYR124, ILE104 | ASN183, ASN254, GLN182, SER299, GLN182 | - |
|  | NSP3 | GLY163, GLN269, TYR268, ASP164, GLU167, PRO258 | GLU167 | - |
|  | NSP12 | ASP623, THR556, ARG624 ARG555, LYS551, ARG553, PRO620, LYS621 | ARG553, ARG624, THR556, ASP623 | - |
|  | NSP13 | ARG443, LYS288, GLY285, THR286, GLY287, GLY538 | GLY285, THR286, GLY287, ARG443, GLY538 | - |
|  | Nucleoprotein | ALA264, VAL270, TRP301, LEU291,ARG277, PHE314, PRO309, ALA311, ARG177, LYS261 | ALA311 | - |
|  | ORF3a | ILE37, TRP45, PHE87 | - | - |
|  | ORF9b | ILE19, VAL94, VAL15, LEU52,VAL76, LEU54 | - | - |
| Bisdequalinium chloride | NSP6 | ARG93, TRP97, MET115 | - | - |
|  | NSP8 | ALA14, ALA18, VAL44 | - | - |
|  | ORF3a | ILE37, PHE87 | - | - |
|  | ORF6 | ILE11, LEU35, ILE36, ILE32 | - | - |
|  | ORF9B | LEU52, LEU54, ILE19, ILE44 | - | - |
| Midostaurin | Membrane | VAL51, LIE71, ILE67, THR68, LEU48 | THR68 | - |
|  | NSP2 | ASN92, GLY173, GLU172, ILE216, ALA225, PRO13 | GLU172, GLY173 | - |
|  | NSP5 | LEU141, CYS145, ASN142 |  | - |
|  | NSP12 | ARG553, LYS551, ASP760, ASP623, ARG624, THR556 | LYS551, THR556 | - |
|  | NSP14 | ALA187, ASN252, ASP273, ASP90,GLY93 | GLY93, ASN252 | - |
| Temoporfin | NSP6 | ARG93, ILE94, ALA119, TRP97, MET115 | - | - |
|  | NSP9 | PHE41, THR68, GLU69, LYS93, ILE66 | GLU69, THR68 | - |
|  | Nuceloprtoein | ARG259, PHE314, LYS261, ALA311, VAL270, ALA264, TRP301, ARG277, ARG177 | ARG259 | - |
|  | ORF3a | VAL88, VAL50, LEU46, ILE47,SER92, LEU95, LEU108, LEU96, LEU95 | SER92, LEU95 | - |
|  | ORF6 | HIS3, LEU40, SER43, GLU46, PHE7 | HIS3, SER43 | - |
| Titilizad | Membrane | ARG63, ILE67, CYS55, VAL51, THR52, TRP49, ALA72, ILE71, TRP22, LEU48 | THR52 | - |
|  | NSP16 | MET6929, LEU6898, ASP6897, TYR6930, SER7000 | SER7000, TYR6930, ASP6897 | - |
|  | ORF3a | LEU108, THR89, VAL112, LEU85, VAL88, VAL50 | VAL88, THR89 | - |
|  | ORF6 | ILE36, LEU40, PHE7, LYS48 | ILE36, PHE7 | - |
|  | ORF8 | PRO36, LEU22, ASP34, CYS20 | LEU22, ASP34 | - |
| Venetoclax | NSP3 | TYR264, ASP164, PRO248, TYR268, PRO299, GLU167 | GLU167 | - |
|  | NSP9 | PHE57, VAL42, ARG40, PHE41, ASN99, ASN96, GLY94, LEU98, SER6 | SER6, ASN96, ASN99, SER6, GLY94, ARG40 | - |
|  | NSP13 | ASP315, LYS288, ALA316, LYS320, ALA312, SER539, ARG178, SER535 | ARG178, SER539, SER535 | - |
|  | ORF8 | LEU22, VAL117, CYS20, SER21, GLN18, HIS17 | GLN18, SER21 | - |
|  | ORF9b | VAL94, ILE44, VAL15, ILE19,LEU52, VAL76, LEU54, ALA57, ASN55, PHE69 | ALA57, ASN55 | - |
| Paritaprevir | NSP2 | ALA357, PRO181, ASN328, ASN254, ALA302, GLN182 | GLN182, ASN254, ASN328 | - |
|  | Nucleoprotein | ALA311, GLY175, ARG177, TRP301, LEU291, VAL270, ARG277, PHE286,ALA308, PHE274 | GLY175, ARG177, ARG177, ARG177, TRP301, GLU174 | - |
|  | ORF6 | SER43, MET1, LEU4, PHE7 | SER43 | - |
|  | ORF9b | ALA57, ASN55, VAL15, LEU54, VAL76, LEU52, VAL96, VAL94 | ALA57, ASN55 | - |
| Zorubicin | NSP2 | ASN254, TYR179, ALA357, PRO181, GLN182, SER301, GLU359, ALA302 | ASN254, GLU359, SER301, ALA302 | - |
|  | NSP9 | ARG40, MET13, PHE41, LYS93 | LYS93 | - |
|  | NSP16 | LYS6968, LYS6844, TYR6930, ASN6996, SER6999, LYS6935, SER7000, GLU6971, TYR6828, CYS6823, LEU6825 | LYS6844, LYS6844, TYR6930, LYS6968, SER7000 | - |
|  | ORF8 | SER21, HIS17, CYS20, GLU19, GLN18 | GLN18, GLU19, CYS20, SER21 | - |
| Suramin | NSP2 | ALA302, ASN133, GLN182, SER358, PHE356, ASN328, ASN254, PHE329, LYS187, TYR189 | LYS187, TYR189, ASN328, SER358, ALA302,LYS187 | - |
|  | NSP5 | THR190, ARG188, ASN53, TYR54, ARG40, LEU50, GLN189, GLU143, HIS41, GLU166, HIS163, SER144, CYS145 | ARG40, TYR54, GLY143, CYS145, HIS163, GLU166, GLN189, THR190, THR190, HIS41, ASN53, ARG188 | - |
|  | NSP9 | SER106, THR110, GLN12, TYR32, LEU10, ALA108, LEU113, VAL77, GLN114, LEU104 | GLN12, TYR32, VAL77, SER106, THR110, GLN114 | - |
|  | Nulcleoprotien | THR245, GLN229, ARG14, PRO13, LYS261, ARG177, PHE315, TYR333, GLN9, TRP330, ARG259, PHE274 | GLN9, ARG14, ARG177, GLN229, :THR245, TYR333, TRP3309 | - |
| Conivaotan | Membrane | ALA72, TRP49, ILE67, LEU48, MET75, LEU45, LYS41 | LYS41 | - |
|  | Nucleoprotein | Phe286, ARG277, LEU291, TRP301,VAL270, ARG177, LYS261, VAL158, ALA156 | - | - |
|  | ORF6 | ILE14, ILE36, ILE11, LEU40 | - | - |
| Lumacaftor | NSP14 | ASN266, ASP273, GLN254, LEU253, PE146 | GLN254, ASN266 | - |
|  | NSP15 | ILE328, GLU327, SER329, THR326, ASN75 | SER329, ASN75 | - |
|  | Spike | TYR505, ARG403, GLY496, GLN493, TYR449, SER494 | GLN493, SER494, GLY496, ARG403 | - |
| Naldemedine | NSP3 | PRO248, TYR268, TYR264, ASP164, TYR273, LEU162, GLY163 | TYR273, PRO248, TYR268 | - |
|  | NSP16 | PRO6878, PRO6932, ASP6928, LYS6968, LYS6844, SER6999, LYS6935 | LYS6844, LYS6968, LYS6968, ER6999, ASP6928 | - |
|  | ORF9b | VAL96, SER53, LEU52, VAL94, LEU21, ILE44, VAL15, VAL76, LEU54, LEU52 | SER53 | - |
| Nilotinib | Membrane | ARG63, VAL51, ILE67, CYS55, LEU48, ALA74, ALA72, TRP49, TRP46, ILE71, LEU45, MET75 | TRP49, TRP46 | - |
|  | ORF3a | LEU83, LEU86, PHE87, VAL90, TYR91, LEU41, ILE37, TRP45 | - | - |
|  | Spike | LYS417, ARG403, TYR453,PHE497,GLY496, SER494, GLN493, TYR505, TYR495 | GLN493, GLY496, E:GLN493, SER494 | - |
| Tasosartan | NSP4 | THR495, MET458, ILE494 | MET458, THR495 | - |
|  | NSP5 | THR25, LEU27, CYS145, GLU166 | GLU166, CYS145 | - |
|  | NSP15 | TYR343, HIS250, LEY346, HIS235 | HIS235 | - |
| Zafirlukast | Membrane | TRP49, THR52, ILE71, LEU48, ILE67, VAL51, TRP46, TRP22 | TRP22, THR52 | - |
|  | NSP3 | ASP164, TYR273, LEU162, TYR264, PRO248, PRO247, PRO299, GLY266 | GLY266, TYR273, ASP164 | - |
|  | NSP13 | LYS288, GLN537, ARG443, SER289, ALA316, ALA312, GLY538, SER539 | SER289, ARG443, GLY538, SER539, LYS288, GLY538 | - |

**Table S6.** Gene enrichment analysis for Biological process, Cellular component and Molecular function of genes associated with top screened drugs interacting with multiple SARS-CoV2 targets. It represents the enrichment FDR, number of genes involved in a particular pathway, the pathways involved and the list of genes associated with the pathway from the list of input genes.

| **Gene Ontology** | **Enrichment FDR** | **nGenes** | **Pathway Genes** | **Fold Enrichment** | **Pathway** | **Genes** |
| --- | --- | --- | --- | --- | --- | --- |
| **Biological Process** | 1.07E-12 | 8 | 82 | 101.0909091 | Vasoconstriction | HTR2A ADRA1A AVPR2 HTR1B AGTR1 ADRA1B ADRA1D HTR1D |
|  | 3.99E-15 | 10 | 106 | 97.75300172 | Phospholipase C-activating G protein-coupled receptor signaling pathway | OPRK1 HTR2A OPRM1 OPRD1 ADRA1A AGTR1 ADRA1B FSHR ADRA1D P2RY2 |
|  | 6.28E-13 | 9 | 133 | 70.11756664 | Regulation of tube diameter | HTR2A ADRA1A AVPR2 HTR1B AGTR1 ADRA1B ADRA1D P2RY2 HTR1D |
|  | 6.28E-13 | 9 | 133 | 70.11756664 | Blood vessel diameter maintenance | HTR2A ADRA1A AVPR2 HTR1B AGTR1 ADRA1B ADRA1D P2RY2 HTR1D |
|  | 6.28E-13 | 9 | 134 | 69.59430122 | Regulation of tube size | HTR2A ADRA1A AVPR2 HTR1B AGTR1 ADRA1B ADRA1D P2RY2 HTR1D |
|  | 7.21E-14 | 11 | 243 | 46.90534979 | Adenylate cyclase-modulating G protein-coupled receptor signaling pathway | OPRK1 OPRM1 OPRD1 ADRA1A AVPR2 HTR1B PRKCA ADRA1B FSHR ADRA1D HTR1D |
|  | 6.31E-11 | 9 | 241 | 38.69558657 | Positive regulation of ERK1 and ERK2 cascade | ABL1 HTR2A OPRM1 PDGFRB ADRA1A KDR PDGFRA PRKCA FSHR |
|  | 3.98E-14 | 13 | 465 | 28.96852395 | Positive regulation of MAPK cascade | OPRK1 ABL1 HTR2A OPRM1 PDGFRB ADRA1A FLT3 KDR PDGFRA PRKCA ADRA1B FSHR ADRA1D |
|  | 5.32E-11 | 10 | 361 | 28.70309746 | Regulation of cytosolic calcium ion concentration | ABL1 HTR2A OPRM1 ADRA1A PDGFRA HTR1B AGTR1 ADRA1B BCL2 ADRA1D |
|  | 6.28E-13 | 13 | 646 | 20.85195609 | Regulation of MAPK cascade | OPRK1 ABL1 HTR2A OPRM1 PDGFRB ADRA1A FLT3 KDR PDGFRA PRKCA ADRA1B FSHR ADRA1D |
|  | 5.03E-11 | 12 | 719 | 17.29371602 | Cellular ion homeostasis | CFTR ABL1 HTR2A OPRM1 ADRA1A PDGFRA HTR1B AGTR1 ADRA1B BCL2 ADRA1D P2RY2 |
|  | 6.28E-13 | 14 | 839 | 17.29028064 | Ion homeostasis | CFTR ABL1 HTR2A OPRM1 ADRA1A AVPR2 KDR PDGFRA HTR1B AGTR1 ADRA1B BCL2 ADRA1D P2RY2 |
|  | 7.01E-13 | 14 | 882 | 16.44733045 | Cellular chemical homeostasis | CFTR OPRK1 ABL1 HTR2A OPRM1 ADRA1A PDGFRA HTR1B AGTR1 ADRA1B FSHR BCL2 ADRA1D P2RY2 |
|  | 2.50E-12 | 14 | 979 | 14.81771752 | Cellular homeostasis | CFTR OPRK1 ABL1 HTR2A OPRM1 ADRA1A PDGFRA HTR1B AGTR1 ADRA1B FSHR BCL2 ADRA1D P2RY2 |
|  | 4.81E-11 | 13 | 944 | 14.269453 | MAPK cascade | OPRK1 ABL1 HTR2A OPRM1 PDGFRB ADRA1A FLT3 KDR PDGFRA PRKCA ADRA1B FSHR ADRA1D |
|  | 4.54E-12 | 14 | 1028 | 14.11142554 | Positive regulation of intracellular signal transduction | OPRK1 ABL1 HTR2A OPRM1 PDGFRB ADRA1A FLT3 KDR PDGFRA PRKCA ADRA1B FSHR BCL2 ADRA1D |
|  | 1.67E-13 | 16 | 1264 | 13.11622555 | Chemical homeostasis | CFTR OPRK1 ABL1 HTR2A OPRM1 ADRA1A AVPR2 KDR PDGFRA HTR1B AGTR1 ADRA1B FSHR BCL2 ADRA1D P2RY2 |
|  | 4.91E-11 | 15 | 1569 | 9.906135929 | G protein-coupled receptor signaling pathway | OPRK1 HTR2A OPRM1 PDGFRB OPRD1 ADRA1A AVPR2 HTR1B AGTR1 PRKCA ADRA1B FSHR ADRA1D P2RY2 HTR1D |
|  | 1.08E-13 | 18 | 1911 | 9.759954331 | Homeostatic process | CFTR OPRK1 ABL1 HTR2A OPRM1 ADRA1A FLT3 AVPR2 KDR PDGFRA HTR1B AGTR1 PRKCA ADRA1B FSHR BCL2 ADRA1D P2RY2 |
|  | 5.01E-11 | 20 | 4258 | 4.866988343 | Regulation of biological quality | CFTR OPRK1 ABL1 HTR2A OPRM1 OPRD1 ADRA1A FLT3 AVPR2 KDR PDGFRA HTR1B AGTR1 PRKCA ADRA1B FSHR BCL2 ADRA1D P2RY2 HTR1D |
|  |  |  |  |  |  |  |
| **Cellular Component** | 4.64E-05 | 2 | 4 | 518.0909091 | Spine apparatus | OPRM1 OPRD1 |
|  | 4.64E-05 | 2 | 4 | 518.0909091 | Serotonin receptor complex | HTR2A HTR1B |
|  | 4.64E-05 | 2 | 4 | 518.0909091 | G protein-coupled serotonin receptor complex | HTR2A HTR1B |
|  | 2.80E-09 | 6 | 70 | 88.81558442 | Integral component of presynaptic membrane | OPRK1 HTR2A OPRM1 OPRD1 ADRA1A HTR1B |
|  | 4.43E-09 | 6 | 79 | 78.69735328 | Intrinsic component of presynaptic membrane | OPRK1 HTR2A OPRM1 OPRD1 ADRA1A HTR1B |
|  | 1.84E-06 | 5 | 119 | 43.53705118 | Integral component of postsynaptic membrane | OPRK1 HTR2A OPRM1 OPRD1 ADRA1A |
|  | 1.94E-06 | 5 | 125 | 41.44727273 | Intrinsic component of postsynaptic membrane | OPRK1 HTR2A OPRM1 OPRD1 ADRA1A |
|  | 1.64E-07 | 6 | 151 | 41.17278748 | Presynaptic membrane | OPRK1 HTR2A OPRM1 OPRD1 ADRA1A HTR1B |
|  | 1.64E-07 | 6 | 153 | 40.63458111 | Integral component of synaptic membrane | OPRK1 HTR2A OPRM1 OPRD1 ADRA1A HTR1B |
|  | 2.38E-07 | 6 | 167 | 37.22808928 | Intrinsic component of synaptic membrane | OPRK1 HTR2A OPRM1 OPRD1 ADRA1A HTR1B |
|  | 1.30E-05 | 6 | 360 | 17.26969697 | Membrane raft | HTR2A OPRM1 OPRD1 ADRA1A KDR ADRA1B |
|  | 1.30E-05 | 6 | 360 | 17.26969697 | Membrane microdomain | HTR2A OPRM1 OPRD1 ADRA1A KDR ADRA1B |
|  | 1.84E-06 | 7 | 422 | 17.18785006 | Receptor complex | HTR2A PDGFRB FLT3 KDR PDGFRA HTR1B FSHR |
|  | 2.25E-05 | 6 | 401 | 15.50396735 | Synaptic membrane | OPRK1 HTR2A OPRM1 OPRD1 ADRA1A HTR1B |
|  | 2.46E-05 | 7 | 674 | 10.76153224 | Dendrite | OPRK1 ABL1 HTR2A OPRM1 OPRD1 HTR1B HTR1D |
|  | 2.46E-05 | 7 | 676 | 10.72969338 | Dendritic tree | OPRK1 ABL1 HTR2A OPRM1 OPRD1 HTR1B HTR1D |
|  | 2.69E-14 | 18 | 1881 | 9.915615485 | Integral component of plasma membrane | CFTR OPRK1 HTR2A OPRM1 PDGFRB OPRD1 ADRA1A FLT3 AVPR2 KDR PDGFRA HTR1B AGTR1 ADRA1B FSHR ADRA1D P2RY2 HTR1D |
|  | 2.92E-14 | 18 | 1965 | 9.491741846 | Intrinsic component of plasma membrane | CFTR OPRK1 HTR2A OPRM1 PDGFRB OPRD1 ADRA1A FLT3 AVPR2 KDR PDGFRA HTR1B AGTR1 ADRA1B FSHR ADRA1D P2RY2 HTR1D |
|  | 2.26E-05 | 9 | 1323 | 7.048855906 | Plasma membrane region | CFTR OPRK1 HTR2A OPRM1 PDGFRB OPRD1 ADRA1A HTR1B ADRA1B |
|  | 2.47E-05 | 11 | 2279 | 5.001316367 | Cell junction | OPRK1 ABL1 HTR2A OPRM1 PDGFRB OPRD1 ADRA1A KDR PDGFRA HTR1B HTR1D |
|  |  |  |  |  |  |  |
| **Molecular Function** | 2.81E-08 | 3 | 3 | 1036.181818 | Alpha1-adrenergic receptor activity | ADRA1A ADRA1B ADRA1D |
|  | 2.88E-05 | 2 | 3 | 690.7878788 | Platelet-derived growth factor-activated receptor activity | PDGFRB PDGFRA |
|  | 1.87E-07 | 3 | 5 | 621.7090909 | Vascular endothelial growth factor binding | PDGFRB KDR PDGFRA |
|  | 3.36E-07 | 3 | 6 | 518.0909091 | Alpha-adrenergic receptor activity | ADRA1A ADRA1B ADRA1D |
|  | 5.35E-07 | 3 | 7 | 444.0779221 | Vascular endothelial growth factor-activated receptor activity | FLT3 KDR PDGFRA |
|  | 1.68E-06 | 3 | 10 | 310.8545455 | Adrenergic receptor activity | ADRA1A ADRA1B ADRA1D |
|  | 2.13E-06 | 3 | 11 | 282.5950413 | G protein-coupled opioid receptor activity | OPRK1 OPRM1 OPRD1 |
|  | 2.39E-06 | 3 | 12 | 259.0454545 | Amine binding | HTR2A HTR1B HTR1D |
|  | 2.39E-06 | 3 | 12 | 259.0454545 | Serotonin binding | HTR2A HTR1B HTR1D |
|  | 2.25E-10 | 6 | 49 | 126.8794063 | G protein-coupled amine receptor activity | HTR2A ADRA1A HTR1B ADRA1B ADRA1D HTR1D |
|  | 2.86E-05 | 3 | 28 | 111.0194805 | Neuropeptide binding | OPRK1 OPRM1 OPRD1 |
|  | 1.87E-07 | 6 | 157 | 39.59930515 | G protein-coupled peptide receptor activity | OPRK1 OPRM1 OPRD1 AVPR2 AGTR1 FSHR |
|  | 1.87E-07 | 6 | 161 | 38.61547149 | Peptide receptor activity | OPRK1 OPRM1 OPRD1 AVPR2 AGTR1 FSHR |
|  | 2.39E-06 | 5 | 137 | 37.81685468 | Transmembrane receptor protein tyrosine kinase activity | ABL1 PDGFRB FLT3 KDR PDGFRA |
|  | 4.05E-06 | 5 | 156 | 33.21095571 | Protein tyrosine kinase activity | ABL1 PDGFRB FLT3 KDR PDGFRA |
|  | 4.05E-06 | 5 | 156 | 33.21095571 | Transmembrane receptor protein kinase activity | ABL1 PDGFRB FLT3 KDR PDGFRA |
|  | 1.58E-12 | 14 | 1026 | 14.13893319 | G protein-coupled receptor activity | OPRK1 HTR2A OPRM1 PDGFRB OPRD1 ADRA1A AVPR2 HTR1B AGTR1 ADRA1B FSHR ADRA1D P2RY2 HTR1D |
|  | 4.07E-15 | 18 | 1671 | 11.1617431 | Transmembrane signaling receptor activity | OPRK1 ABL1 HTR2A OPRM1 PDGFRB OPRD1 ADRA1A FLT3 AVPR2 KDR PDGFRA HTR1B AGTR1 ADRA1B FSHR ADRA1D P2RY2 HTR1D |
|  | 1.92E-14 | 18 | 1940 | 9.614058107 | Signaling receptor activity | OPRK1 ABL1 HTR2A OPRM1 PDGFRB OPRD1 ADRA1A FLT3 AVPR2 KDR PDGFRA HTR1B AGTR1 ADRA1B FSHR ADRA1D P2RY2 HTR1D |
|  | 1.92E-14 | 18 | 1940 | 9.614058107 | Molecular transducer activity | OPRK1 ABL1 HTR2A OPRM1 PDGFRB OPRD1 ADRA1A FLT3 AVPR2 KDR PDGFRA HTR1B AGTR1 ADRA1B FSHR ADRA1D P2RY2 HTR1D |

Reference:

1.Wang Z, Pan H, Sun H, Kang Y, Liu H, Cao D, Hou T. fastDRH: a webserver to predict and analyze protein-ligand complexes based on molecular docking and MM/PB(GB)SA computation. Brief Bioinform. 2022; 23(5): bbac201.
