# Supplementary material for: Polypharmacology guided drug repositioning approach for SARS-CoV2
Source: PLoS One. 2023 Aug 9;18(8):e0289890. doi: 10.1371/journal.pone.0289890 (PMC10411734; doi:10.1371/journal.pone.0289890)
Supplement: Attachment — Submitted filename: Response to reviewers.docx [file pone.0289890.s003.docx]

**19^th^ July 2023**

Dr. Chandrabose Selvaraj

Academic Editor, PLOS One

**Sub:** Submission of the revised manuscript entitled “*Polypharmacology Guided Drug Repositioning Approach for SARS-CoV2”* (PONE-D-23-10719) by Jamir *et. al.,*

Dear Professor Chandrabose,

Thank you for your communication regarding the reviewer’s comment and considering our manuscript our revised manuscript. Current second revision contain makes an attempt to satisfactorily address the queries raised by the reviewers. The changes are incorporated in the manuscript and highlighted. You please find the point-wise response below,

**Reviewer #1**

**Query 1**. **Although authors have responded to my request, docking studies without the support of MD simulations combined with end-point or alchemical free energy calculations are not enough to provide profound and strong computational drug repurposing or drug design results.**

**Answer.** While we appreciate the concern of the referee regarding the rigorous quantification of the binding energy some of our practical concerns, we would like to bring forth our apprehensions as given in the earlier response to the query in revision 1.

While exhaustive MD simulations are quite insightful, the time step required for each system is different and that makes it not very practical to employ a uniform time step. Further, we would like to bring to the attention of the revered referee that proteins and ligands considered in this study are of various size and getting a consistent and comparable trajectory will be a challenge. In addition to the three docking procedures that we have adopted, we chose to search for a reliable alternative to the docking method. Thus, we employed MM-PB/GBSA analysis using the web server <http://cadd.zju.edu.cn/fastdrh/submit> [*Briefings in Bioinformatics*, *23*(5), bbac201], while these approaches are far from traditional MD trajectory driven analogs, the MM-PB/GBSA obtained are in good agreement with the docking results.

Further, we also would like to mention that the focus of the current manuscript is on polypharmacology and drug repurposing, which are essentially dependent on qualitative trends. Given the consensus observed among different docking procedures and the comprehensive nature of the complexes under investigation, we believe that incorporating MD simulations may not provide significantly deeper insights. In our opinion, this study adopts the most exhaustive approach to drug repurposing by utilizing polypharmacology principles, aided by therapeutic indication analysis and docking studies. This strategy has successfully identified drugs that target multiple proteins and pathways critical for enhancing drug potency against virus variants.

**Reviewer #3:**

**MINOR REVISION**

**I would like to submit following comments in connection with the manuscript titled “Polypharmacology Guided Drug Repositioning Approach for SARS-CoV2”.**

**Abstract**

**Query 1.** **In lines no 30-40, As a result, the top 10 drugs ……….. biological process. Can be changed into “As a result, the top 10 drugs were found to have therapeutic indications for cancer, pain, neurological disorders, viral and bacterial diseases.**

**Answer.** We thank the reviewer for taking time to go through the manuscript and providing valuable suggestions. As suggested, the line “As a result, the top 10 drugs ……….. biological process” have been changed into “As a result, the top 10 drugs were found to have therapeutic indications for cancer, pain, neurological disorders, viral and bacterial diseases.

**Query** **2**. **As drug resistance is one of the major challenges in antiviral drug discovery, polypharmacology and network pharmacology approaches were employed in this study to identify drugs interacting with multiple targets and drugs such as dihydroergotamine, ergotamine, bisdequalinium chloride, midostaurin, temoporfin, tirilazad, and venetoclax were identified among the multi-targeting drugs.**

**Answer.** The sentence has been modified as per the suggestion of the reviewer.

**Query** **3. Further, a pathway analysis of the genes related to the multi-targeting drugs was carried out which provides insights into the mechanism of drugs and identifying targetable genes and biological pathways involved in SARS-CoV2.**

**Answer.** The sentence has been modified as per the suggestion of the reviewer.

**Table**

**Query** **4. Table 3a, under Spike protein heading, HIV-1 infections can be changed into HIV-1 infection.**

**Answer.** Table 3a, under Spike protein heading, HIV-1 infections have be changed into HIV-1 infection.

**Results**

**Query** **5. In line no 441, According to a study reported by Deng et al., 2021, ……….plasma membrane [74]. Can be changed into “According to a study reported by Deng et al. (2021),, ………. and directs fusing of the viral membrane with the host plasma membrane [74].”**

**Answer. The line,** “According to a study reported by Deng et al., 2021, ……….plasma membrane [74]. have been changed into “According to a study reported by Deng et al. (2021),, ………. and directs fusing of the viral membrane with the host plasma membrane [74].”

**Query** **6. In lines no 454-456, This indicates the significance………..SARS-CoV-2 infection [71, 72] can be changed into “This indicates the significance………..SARS-CoV-2 infection [71, 72].”**

**Answer.** The line “This indicates the significance………..SARS-CoV-2 infection [71, 72] have been changed into “This indicates the significance………..SARS-CoV-2 infection [71, 72].”

**Discussion**

**Query** **7. In line no 513, The docking score of the top 10 FDA approved………. all the three methods. Can be changed into “The docking scores of the top 10 FDA approved………. all the three methods.”**

**Answer.** The line “The docking score of the top 10 FDA approved………. all the three methods have been into “The docking scores of the top 10 FDA approved………. all the three methods.”

We hope that our revision is satisfactory for you. We remain with many thanks to the reviewers for their constructive suggestions and you for the prudent handling of the manuscript.

With Regards,

G. Narahari Sastry
